# 2402. Evaluating Clinical Outcomes of Adjunctive Beta-Lactam Use in Vancomycin-Resistant *Enterococcus* Infective Endocarditis

**DOI:** 10.1093/ofid/ofad500.2022

**Published:** 2023-11-27

**Authors:** Ebrahim H Khan, Zachary A Yetmar, Omar M Abu Saleh, Maryam Mahmood

**Affiliations:** Mayo Clinic, Rochester, Minnesota; Mayo Clinic, Rochester, Minnesota; Mayo Clinic Rochester, Rochester, Minnesota; Mayo Clinic, Rochester, Minnesota

## Abstract

**Background:**

Vancomycin resistant enterococcus (VRE) infective endocarditis (IE) is associated with poor outcomes. There are limited data to guide the antimicrobial choice and duration of therapy. Several *in vitro* and limited clinical studies demonstrate the synergistic effect of β-lactams with other agents, particularly daptomycin, in this setting. We sought to compare clinical outcomes in patients with VRE IE treated with adjunctive β-lactam therapy.

**Methods:**

This was an IRB-approved, retrospective, single-center cohort study. The study included patients ≥18 years of age diagnosed with VRE IE via modified Duke criteria from 2011-2021. Data were collected by electronic medical record review. Descriptive statistics were obtained. The primary outcome was 90-day treatment failure compared between those receiving and not receiving adjunctive β-lactams. Treatment failure was defined as recurrence of bloodstream infection (BSI) or death within 90 days of diagnosis. Failure-free survival was analyzed by Kaplan-Meier curve with log-rank test.

**Results:**

A total of 32 cases were identified. 12 received adjunctive β-lactams. Mean age was 67 years, and 71% were male. 13 (41%) had a prosthetic valve and 6 (19%) had a CIED (Table 1). A majority (78%) had *E. faecium* IE. Mean duration of bacteremia was 9 days (Table 2). About one-third had treatment failure with 7 deaths and 4 BSI recurrences. Adjunctive β-lactams included ampicillin (3), cefepime (1), ceftaroline (6) and ceftriaxone (2). There was no statistical difference in failure-free survival in patients treated with adjunctive β-lactam therapy (Figure 1). The results were similar when restricted only to those who received daptomycin with or without adjunctive β-lactam therapy.

**Table 1.**
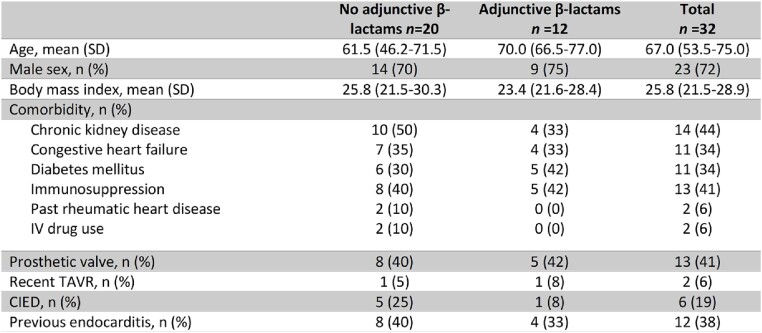
Patient Characteristics

**Table 2.**
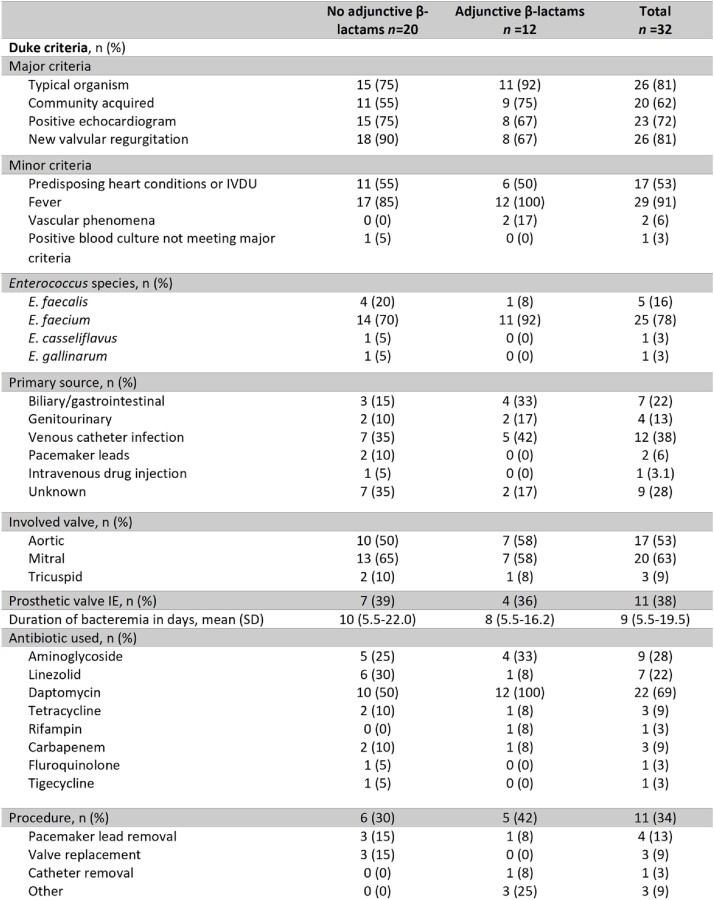
Disease characteristics

Figure 1.

**Figure:**
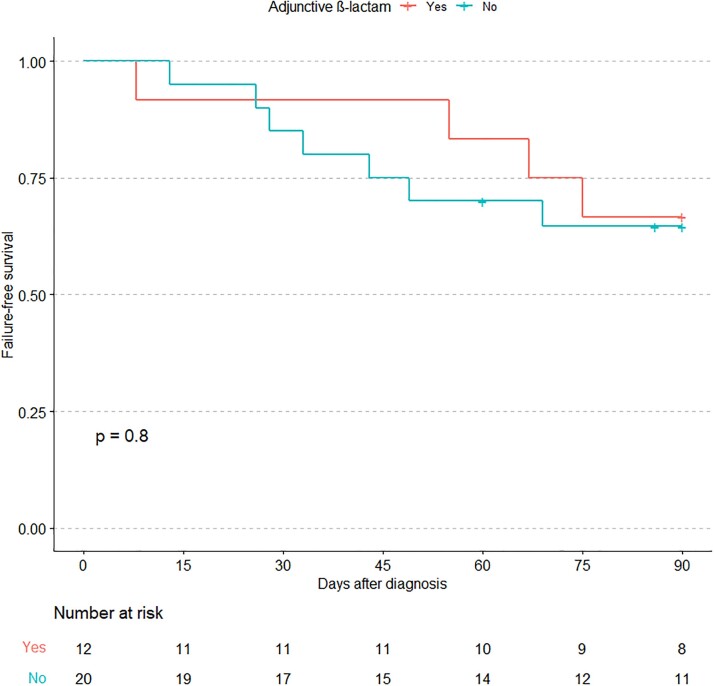

Kaplan-Meier curve comparing failure-free survival between patients who were primary treated with adjunctive β-lactams versus non-adjunctive therapy. The p-value is calculated via the log-rank test.

**Conclusion:**

VRE IE is associated with poor prognosis, including high rates of mortality and relapses. In our experience, adjunctive B-lactam use did not result in improved outcome. Limitations include small sample size, and heterogenous antibiotic use. More prospective randomized data are needed.

**Disclosures:**

**All Authors**: No reported disclosures

